# Depression and somatization in Turkish migrants in Germany: the role of migration-related stressors

**DOI:** 10.3389/fpsyt.2025.1642491

**Published:** 2025-10-14

**Authors:** Bernd Hanewald, Eric Hahn, Tam Thi Minh Ta, Yasemin Elguen, Markus Stingl

**Affiliations:** ^1^ Center for Psychiatry, Justus Liebig University Giessen, Giessen, Germany; ^2^ Department of Psychiatry and Neurosciences, Charité – Universitätsmedizin Berlin, Berlin, Germany

**Keywords:** migration-related stressors, Turkish migrants, acculturation, cross-cultural psychiatry, depression, somatization

## Abstract

**Introduction:**

Migration-related stressors (MRS), such as loss, cultural conflict, and language barriers, are linked to increased psychological distress. This study investigates the impact of MRS on de-pressive symptoms and somatization among Turkish-speaking first-generation migrants in Germany.

**Method:**

In a cross-sectional study, 60 psychiatric outpatients completed standardized measures as-sessing MRS, depressive symptoms (BDI-II), and somatization (PHQ-15). Regression analy-ses controlled for age, gender, and socioeconomic status.

**Results:**

Higher MRS significantly predicted both depressive symptoms and somatization. Somatic complaints were particularly associated with language difficulties, separation experiences, and intergenerational value conflicts.

**Conclusion:**

Findings support the need to integrate cultural humility—defined as clinician self-awareness, openness, and contextual sensitivity—into evidence-based care. Symptom interpretation must consider patients’ migration histories and cultural frameworks. Culturally adapted, person-centered interventions may improve diagnostic accuracy and treatment outcomes for migrant populations.

## Introduction

Germany remains one of the main destinations for migrants in Europe (1, 2). Currently, about one in four residents has a migration background, accounting for 26.0% of the population. Having a migration background is defined as being a person or having at least one parent who was not born with German citizenship. The largest migrant group in Germany originates from Turkey. Historically, between the 1960s and 1970s, approximately 750,000 to 900,000 Turkish guest workers arrived, and today about 2.8 million people of Turkish origin live in Germany (3). These individuals can be distinguished by migration circumstances and generational status, with each generation facing distinct challenges. Although, according to the Federal Commissioner for Integration, integration has improved in general, many institutions are still not adequately adapted to the realities of a diverse society (4). The exact number of first-generation Turkish migrants is difficult to determine, as many have naturalized and are no longer counted as foreigners in statistics.

With regard to mental health, migrants are at elevated risk for psychiatric disorders such as somatoform disorders, substance abuse, depression, and psychosis (5). Against this background, it becomes evident that the Turkish population in Germany is disproportionately affected by specific challenges in accessing mental health services. Turkish migrants in Germany encounter structural barriers - including language difficulties, lack of culturally sensitive services, and financial constraints - as well as non-structural barriers such as stigma, mistrust, and culturally specific expressions of psychological distress, which may lead to misdiagnosis. These factors collectively reduce help-seeking and worsen mental health outcomes among Turkish patients (6–8). Culturally adapted interventions, language-appropriate services, community outreach, and cultural mediators are therefore essential.

## Migration processes and mental health

Migration can entail both protective and adverse effects on mental health. On the positive side, improved socioeconomic conditions and future prospects may enhance well-being (9). On the contrary, migration constitutes a critical life event that involves acculturative stress, often accompanied by physical and psychological problems (10, 11).

Although migration per se is not causally related to mental illness, numerous studies demonstrate a strong association between migration-related stressors (MRS) and psychiatric disorders, including dose-response effects: the greater the number of MRS, the higher the prevalence of mental disorders (9, 12). Stressors may occur before, during, and after migration, including separation from family, loss of familiar lifestyles, loneliness, discrimination, socioeconomic hardship, separation grief, communication difficulties or language barriers, unemployment, unmet expectations, and stigmatization.

A retrospective study of 268 immigrant patients in a psychiatric intensive care unit (13) found that 51.9% experienced acculturation stress, especially men, single individuals, unemployed persons, and those without residence permits. Acculturation stress was associated with psychotic disorders, first psychiatric episodes, and poorer outcomes at discharge. Similarly, Choy et al. (14) showed that marginalization had the most detrimental, and integration the most beneficial effects on mental health.

The nature of stressors differs between first- and second-generation migrants. The first generation faces challenges linked to migration motives, journey, and language acquisition, while the second generation more frequently struggles with identity-related issues, prejudice, and discrimination (15).

Migrants - particularly those who relocate by choice rather than through forced displacement - experience a distinct spectrum of stressors related to their migration. Pre-migration stressors in the context of voluntary migration often involve socioeconomic challenges such as limited educational or occupational opportunities, rather than persecution or war. For example, migrants may depart in search of improved career prospects or educational advancement, yet still face internal pressures such as high expectations or a sense of opportunity loss if those aspirations are unmet (16, 17). Post-migration stressors, emerging after arrival in the host country, frequently include education-occupation mismatch, discrimination, poor working conditions, and acculturative stress - with the latter sometimes described as a fatigue-inducing process marked by language difficulties, loneliness, and cultural disorientation (17).

Empirical studies suggest that while pre-migration pressures in voluntary migrants may derive from personal or socioeconomic motivations, the stressors encountered post-migration often have greater impact on mental health outcomes. Voluntary migrants are reported to experience roughly 50% less acculturative stress than refugees, yet still face substantial challenges such as over-qualification, underemployment, and barriers to social integration, all of which correlate strongly with depression and anxiety (16, 17).

Addressing post-migration stressors - e.g., through recognition of foreign qualifications, culturally sensitive workplace policies, and integration programs - represents a key target for prevention.

## Language as a key factor

Language and communication are central determinants of integration and mental health. Insufficient language skills hinder healthcare access, impair diagnostic accuracy, and weaken therapeutic alliances. Educational attainment also plays a role: the first generation of Turkish migrants, recruited primarily as industrial workers in the 1960s and 1970s, often had limited educational opportunities, a disadvantage that affected subsequent generations. In particular for the first generation, language barriers were a significant obstacle to integration into the education system and the labour market (18, 19). In recent decades, the levels of education and qualifications among migrants have improved. Children and grandchildren of first-generation migrants are increasingly achieving higher educational qualifications. Many younger migrant families strongly emphasize education, and targeted support measures are helping to foster this development.

Limited host-country language proficiency is consistently associated with an increased prevalence and severity of psychiatric disorders, including psychotic, affective, anxiety, and post-traumatic stress disorders (20). In psychiatric care, low language proficiency correlates with poorer treatment outcomes and a greater likelihood of coercive interventions, such as involuntary admission, forced medication, and restraint (21). Studies show that better language skills - more common among women, legally residing migrants, and those from European countries - are linked to later illness onset and shorter treatment duration (22, 23). Systematic reviews confirm that language barriers reduce utilization of mental health services and contribute to underdiagnosis (24, 25). Addressing this modifiable stressor through structured language training and professional interpretation services should be a core component of culturally responsive psychiatric care.

Perceived discrimination is another key MRS (26). According to the 14th Integration Report, one in five immigrants and one in four descendants report personal experiences of discrimination (4). Discrimination in employment, housing, education, or public services contributes to psychological distress, and fear of discrimination further discourages the utilisation of healthcare, particularly for mental illness.

## Migration and susceptibility to illness

The health burden of migration is particularly pronounced when disadvantages in multiple life domains accumulate (27). Many migrants live with low socioeconomic status, limited social networks, and exposure to migration-related stressors, which collectively increase vulnerability (28). Sociodemographic factors such as poverty, limited participation opportunities, and institutional discrimination should be considered alongside interpersonal experiences of prejudice (26).

Numerous studies confirm a higher prevalence of psychiatric disorders among migrants, including somatoform disorders, depression, substance abuse, and psychosis (5, 26, 28–30). Turkish migrants in Germany exhibit elevated vulnerability and lower utilisation of psychiatric services, often resulting in delayed treatment and longer duration (31). Contributing factors include fear of stigmatization, somatically oriented illness concepts, and lack of therapy options in Turkish.

## Culturally influenced illness perceptions and treatment barriers

Cultural and religious beliefs can strongly shape illness concepts in Turkish migrants. In Islam, illness may be interpreted as fate, while supernatural attributions such as spirits (*Cins*) or the *Nazar* (evil eye) remain common (31–35). Folk medicine explanations are prevalent in cases of psychological distress, unclear symptoms, or when other treatments fail and may relieve personal guilt and legitimize illness roles within families and communities (33). Consequently, many first-generation migrants prefer traditional healers (e.g., *Hoca*) over psychiatric care.

During treatment by German doctors, migrants of Turkish origin may not fully understand the doctor’s recommendations and instructions, leading to inadequate treatment adherence and compliance. At the same time, they often present with culturally distinct symptoms, which can lead to misdiagnoses and complicated treatment courses. These perceptions of the illness, combined with language barriers, complicate medical treatment. Turkish patients often attribute illness to external factors and hold high expectations of healthcare providers, creating additional challenges. Addressing these culturally shaped perceptions is therefore crucial for effective treatment (31, 36).

## Summary and study objective

Investigating migration-related stressors among Turkish migrants - particularly first- and second-generation - in Germany is of high relevance given their longstanding presence and distinct sociocultural dynamics. Research shows that first-generation migrants adopting marginalisation or separation acculturation patterns exhibit higher depressive symptoms, while integration correlates with lower burden (7). Separation and marginalization are also associated with reduced health-related quality of life, particularly among descendants, while integration has protective effects (37).

Epidemiological data highlight the substantial mental health burden: In a representative sample of Turkish migrants in Hamburg and Berlin, lifetime prevalence of any mental disorder was 78.8%, with mood disorders (41.9%), anxiety disorders (35.7%), and somatoform disorders (33.7%) being most common. Differences between first- and second-generation migrants were limited, except for higher bipolar disorder rates in the second generation (Dingoyan et al., 2017). In another cohort, first-generation migrants - especially of Turkish origin - showed a higher prevalence of depressive and anxiety symptoms and suicidal ideation compared to native Germans, while the second generation did not show significant differences (38).

Therefore, this study examines migration-related stressors and their relationship to depression and somatization among Turkish-speaking migrants in Germany. The aim is to clarify how such stressors influence the development and course of these disorders, and to derive implications for treatment. By focusing on this population, the study addresses a critical research gap and contributes to culturally sensitive intervention strategies and improved access to care.

## Methods

### Procedure

The study focused on patients of Turkish origin who underwent native language outpatient treatment at a University Hospital for Psychiatry and Psychotherapy.

The study was carried out in the outpatient clinic of a university hospital by a Turkish physician and board-certified specialist in psychiatry and psychotherapy (YE). She worked within a multidisciplinary team comprising a senior psychiatric consultant, additional medical specialists, resident physicians, psychologists and medical assistants, ensuring comprehensive clinical evaluation and care.

The study involved a single interview, randomly occurring during outpatient treatment after diagnosis. Data collected during outpatient treatment, such as essential documentation and patient records, were used whenever possible. Efforts were made to minimize the time burden on each patient. Visual scales and detailed explanations were provided for closed and written questions with predefined answer options to facilitate patient responses. The study population consisted of patients from the outpatient clinic in the Clinic for Psychiatry and Psychotherapy at the University Hospital in Giessen.

Eligible participants were individuals of Turkish origin who were receiving treatment in the psychiatric outpatient clinic, met ICD-10 diagnostic criteria for at least one depressive episode (F32.0–F32.2 or F33.0–F33.2), and provided written informed consent. Exclusion criteria included a clinical judgement of the treating physician that participation might interfere with ongoing treatment or adversely affect the participant’s well-being; inability to provide informed consent or refusal to participate; the presence of severe mental impairment, e.g., intellectual disability, dementia, psychotic disorder or delusional depression, acute intoxication, markedly reduced consciousness, or acute suicidality. The data collection phase occurred between January 2022 and April 2022. Patients who met the inclusion and exclusion criteria were informed about the study through personal conversations with the treating specialist. Those providing written consent were briefed verbally and in writing about the purpose, process, and content of the interview. Patients had the option to terminate the survey at any time without consequences. In the clinic, diagnoses are routinely reviewed, validated, and coded on a quarterly basis; prior to the interview, each diagnosis was reevaluated. All patients included were undergoing ongoing outpatient treatment in the clinic at the time of participation. The interview, which included half an hour for explanation and consent and an hour for questionnaire completion, lasted a total of one and a half hours. Following the interview, of the responses were checked for completeness, and the treating physician evaluated the patients based on their questionnaires. All questionnaires were numbered and stored in locked cabinets in the clinic’s outpatient department. The study was approved by the Ethics Committee of the Medical Faculty of the University of Giessen and was conducted in accordance with the Declaration of Helsinki.

### Instruments

#### Questionnaire on sociodemographics and illness characteristics

Survey on medical history and psychosocial status, including questions on sociodemographics and illness characteristics. It includes inquiries about biography, sociodemographics, social networks, the patient’s illness characteristics, and treatment.

#### Questionnaire on Migration-Related Stressors

The Migration-Related Stressors (MRS) questionnaire was developed by Lujic (31) to assess migration-dependent stress factors. For this study, the questions were reevaluated to determine, through a list of 26 questions, whether the corresponding stress factor is present and the extent of the patient’s suffering.

#### Beck Depression Inventory

The Beck Depression Inventory (BDI) (39) measures the severity of depressive symptoms in individuals aged 13 and above in a clinical setting. Symptoms are described on affective, somatic, and cognitive levels, referring to the past week. The inventory consists of 21 items. We used the Turkish version from Hisli (40).

#### Screening for Somatoform Disorders

The Screening for Somatoform Disorders (SOMS) (41) is used for the classification, quantification, and description of somatoform disorders based on the criteria of Somatization Disorder according to DSM-IV (42) and ICD-10 (43) within the last 2 years. The Turkish version of SOMS is attributed to Erim et al. (44).

#### Migration-related stressors

Different types of MRS were assessed using a list of 25 questions, initially formulated by Lujic (31) to evaluate the treatment of Turkish migrants in Germany (see **Table 1**). Participants were asked whether each specific stressor had occurred (“yes” = 1) or not (“no” = 0) during their migration process.

**Table 1:** Sample characteristics.

Sociodemographics	N (%); M (SD)
*Sex*	
male female	25 (41.7) 35 (58.3)
*Age*	48.3 (11.0)
*Children*	2.2 (1.6)
*Martial status*	
married single / separated / divorced / widowed	44 (73.3) 16 (26.7)
*Level of Education*	
lack of education special school secondary school high school university degree	4 (6.7) 20 (33.3) 27 45.0) 6 (10.0) 3 (5.0)
*Status of employment*	
employed housewife / unemployed / retired	21 (35.0) 39 (65.0)
*Country of birth*	Turkey 48 (80.0) Germany 6 (10.0) Other 6 (10.0)
Acculturation characteristics	
*Age at migration*	23.6 (11.6)
*Reason for migration^a^*	
family Reunion asylum Seeker labor migration educational reason other reason	38 (63.3) 7 (11.7) 9 (15) 2 (3.3) 3 (5.0)
Clinical characteristics	N (%)
*BDI-II*	23.6 (13.25)^a^
*SOMS*	15.3 (11.7) ^a^
*Diagnose^b^*	
Depression (F32.XX; F33.XX) Anxiety(F40.XX ) PTSD (F43.1) somatoform disorder (F45.XX) other	59 (98.3)^c^ 9 (15.0) ^c^ 5 (8.33) ^c^ 6 (10.0) ^c^ 1 (1.7) ^c^

^a^N (SD) ^b^multiple/not applicable answers are possible. ^c^N (%) Diagnoses were rated by a psychiatrist referring to ICD-10 criteria.

Individual item scores from the MRS list were totaled to create a Migration Stressor Quantity (MSQ) index, where higher scores indicated a greater number of perceived stressors. Scores were calculated only when more than 75% of the relevant data were provided.

### Statistical analysis

For the main analysis, hierarchical linear regression models were applied to assess the impact of the quantity of MRS on self-reported depressive symptoms and somatization. In the first step, sociodemographic variables (age, sex, and education level) were included as control variables, followed by the introduction of the quantity of MRS as the primary predictor in the second step. The BDI-II total score and SOMS scores were included as separate outcome variables.

All statistical analyses were conducted using IBM SPSS (Version 26). Values of *p* ≤ 0.05 were regarded as statistically significant, while values of *p* ≤ 0.001 were deemed highly significant. All reported *p*-values are based on two-tailed tests.

## Results

### Demographic-, clinical-, and migration-related sample characteristics

In total, 60 Turkish-origin patients who met the inclusion criteria (having secure residential status and having rated more than 75% of the MRS items) participated in the study, which included 35 women and 25 men. The mean age was 48.32 years. On average, participants had resided in the host country for 24.03 years (SD = 14.8). A notable 93.3% of the participants were first-generation migrants, while 6.7% were born in Germany. Additionally, 43.3% of individuals originated from urban regions, and 56.7% came from rural areas. The average age at the time of migration was 23.58 years. The primary reason for migration was cited as family reunification (63.3%), followed by political (11.7%) and economic (8.3%) reasons, job search (6.7%), and studying (3.3%). Moreover, 73.3% of the participants were married, with an average of 2.2 children. Participants mainly arrived in Germany during their youth, lacking professional qualifications from Turkey, and consequently engaged in unskilled occupations in Germany. They predominantly had two or more F-diagnoses. Regarding somatic comorbidities, 55% of the subjects had at least one somatic comorbidity, with arterial hypertension being the most common (35%), followed by diabetes mellitus (10%).

For further information, see **Table 1**.

### Psychometric characteristics of the instruments utilized

The average intercorrelation (Cronbach’s α) of the total BDI-II items was α = .93, indicating a high internal consistency for the BDI-II. The SOMS questionnaire also demonstrated excellent internal consistency with Cronbach’s α = .918. MSR, with Cronbach’s α = .785, was also considered reliable.

### BDI-II and SOMS

The mean BDI-II total score was 23.6 (SD = 13.25), indicating moderate to severe symptomatology among patients of Turkish origin.

Turkish-origin patients exhibited a strong tendency towards somatization in the screening for somatoform disorders (SOMS). The mean SOMS total score of 15.3 (SD = 11.7) indicates moderate to severe somatization symptomatology. They reported significantly more physical symptoms and symptoms of somatization disorder without an organic cause than the standard sample from the general population (Complaint Index according to DSM-IV and ICD-10 criteria).

Both measures (BDI-II and SOMS) were significantly correlated (r = .506, p <.001), indicating an overlap between depression and somatization in the sample (see **Table 1**).

### Migration-related stressors

**Figure 1:**
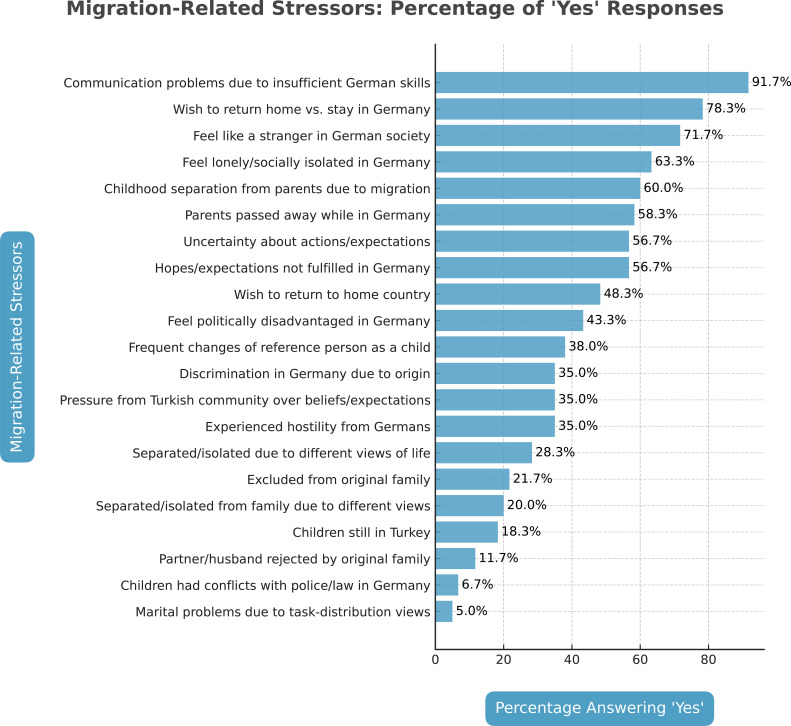
Answers in the Migration Related Stressors Questionnaire (MRS).

Communication problems were most frequently reported regarding migration-related stressors. However, missing family in Turkey, the desire to return, associated indecision, and difficulties adapting to German society were perceived as burdensome. Language issues constituted the greatest burden for 91.7%. More than half of the patients also mentioned prolonged separations from parents during childhood, the death of parents in their absence, loneliness, and feelings of isolation as stressors. Many Turkish-origin patients struggled with a lack of social competence, indicating that they could not assess what others expected of them. Consequently, uncertainties about expectations, unmet personal ambitions, and hopes were identified as stressors. Turkish-origin patients often described experiences of xenophobia and feeling pressured by peers in Turkish society. In contrast, marital problems arising from differing views on task distribution and issues involving children with law enforcement were less frequently reported. (In detail, see **Figure 1**).

### MSQ as predictor and BDI-II and SOMS total scores as outcome variables

Hierarchical linear regression models revealed significant effects of migration stressor quantity (MSQ) as a predictor of the BDI-II and SOMS scores (see **Table 2**).

**Table 2:** Hierarchical regressions^a^ of the MRS-quantity on depressive (BDI-II) and somatization (SOMS) symptoms.

Outcome	M (SD)^b^	B^c^	R^2d^	β^e^	CI (95%)^f^	F^g^	p^h^
BDI-II total	23.6 (13.25)	1.271	.203	.421	0.53 – 2.01	4.760	.001
SOMS total	15.3 (11.7)	1.096	.170	.412	0.43 – 1.76	3.835	.002

^a^Control variables: age, sex and level of education. ^b^Mean (standard deviation).^c^ Unstandardized beta-coefficient. ^d^Coeficient of determination. ^e^Standardized beta-coefficient. ^f^Confidence interval. ^g^F-value. ^h^Level of significance.

The MSQ predictor significantly affects the BDI-II total score (F (3) = 4.76, p = .001, η^2^ = .203), with a greater magnitude of experienced MSR associated with a higher burden of depressive symptoms.

Regarding somatization, the MSQ significantly predicted SOMS values (F (3)=3.835, p=.002, η^2^ = .170), indicating that greater experienced MSR is associated with a higher level of somatization.The sociodemographic factors of *age*, *gender*, and *level of education, which were used* as control variables, had no substantial effect on the results of the regression analyses (all p >.005).

## Discussion

The present study systematically examined migration-related stressors (MRS) and their mental health impact in a sample of migrants treated in a psychiatric hospital setting. Several central findings stand out. First, a very high proportion of patients reported language-related difficulties, with 91.7% indicating that communication barriers significantly affected their ability to interact with healthcare professionals. In addition, more than 78% expressed a desire to return to their country of origin, 63% reported feelings of social isolation in Germany, 60% had been separated from parents due to migration, and 58% had experienced the death of a close family member. Participants also reported uncertainties regarding their future expectations, and more than half indicated that their migration-related expectations had not been fulfilled. These figures illustrate the cumulative burden of MRS and highlight the relevance of acculturative stress as a central determinant of psychological well-being in this population. In light of the citizenship law reforms in Germany enacted in June 2024 - facilitating shorter residency requirements and permitting mulitple citizenship - the relevance of MRS is expected to further increase (4). In our study, participants exhibited moderate to severe levels of both depression (BDI-II) and somatization (SOMS), with a significant correlation indicating substantial symptom overlap.

Second, we found a strong association between MRS and depressive symptomatology. Most notably, there was a dose-response relationship between the number of stressors and depressive symptom severity, suggesting that the accumulation of migration-related stressors is directly linked to an increased risk of depression. This finding is consistent with previous research, which has described a ‘risk accumulation’ model in migrant mental health, where the number and intensity of MRS, rather than a single factor, predict psychiatric outcomes (9, 12).

Third, our study underscores the role of somatization. Migrants with higher levels of MRS reported significantly more somatic symptoms, and we observed a positive correlation between somatization and the number of migration-related stressors. This is particularly relevant in the context of somatic hospitals, where somatization often leads to repeated medical consultations and potentially unnecessary diagnostic procedures. Our study confirmed, as previously established by Erim et al. (8), that somatic complaints among Turkish individuals often express underlying psychological problems. Migration-related stressors (MRS) can further amplify this tendency. In our sample of Turkish patients, MRS plays a central role in the development of psychological disorders, particularly in depressive illnesses and tendencies toward somatization. To support this, Bengi-Arslan et al. (6) found that somatic symptoms such as “tightness” were prevalent among Turkish immigrants and often indicative of underlying psychological distress. These findings highlight the need for comprehensive assessment and culturally sensitive approaches in the treatment of Turkish migrants. Therefore, it is essential to develop a contextualized bio-psycho-social model of illness that considers cultural specificities in treating patients with Turkish background. In addition to assessing individual symptoms, targeted inquiry into MRS and their integration into treatment planning should be prioritized. MRS can cause or exacerbate diseases, which is why a targeted approach to dealing with these stress factors and reducing the resulting burdens is crucial to improving the mental health of Turkish patients.

When compared to previous studies, our findings show both similarities and differences. The high prevalence of language barriers (91.7%) is in line with earlier research demonstrating that linguistic difficulties are among the most consistent predictors of poor mental health outcomes and reduced treatment satisfaction among migrants. A systematic review published by Pandey et al. (45) found that limited proficiency in the host country’s language significantly hinders immigrants’ access to healthcare services. Language barriers have consistently been identified as obstacles to seeking, accessing, and utilizing mental health services. Research in the United States indicates that immigrants from regions such as Asia, Latin America, and Africa use mental health services at lower rates compared to non-immigrants, despite having similar or greater needs. Structural barriers, including language difficulties, contribute to this underutilization (46). The multi-level barriers migrants face in accessing mental health services emphasize that language barriers not only impede service utilization but also exacerbate mental health issues due to increased stress and social isolation (47).

Likewise, the high frequency of social isolation (63%) and the expressed wish to return to the home country (78%) are consistent with evidence that disrupted social networks and ambiguous belonging are central contributors to acculturative stress. These findings align with broader evidence: among Sudanese voluntary migrants in the United Arab Emirates, homesickness was significantly linked to psychological distress, depressive and anxiety symptoms - especially among women, the unemployed, and unmarried individuals, whereas longer residence and higher age mitigated these effects (48). Furthermore, first-generation migrants in the U.S. report lower social support and poorer health, and, unlike non-migrants, do not reap health benefits from social networks. This highlights how social isolation can profoundly impact migrant well-being (49). Together, these results reinforce that migration is not only a life-changing decision but a deeply personal and emotional process that can challenge identity, belonging, and mental health.

The dose-response relationship between stressors and depression observed in our study has also been described in other migrant groups. Turkish-speaking migrants who reported greater difficulties adapting to German society, alongside experiences of discrimination and unmet expectations, were more likely to suffer from depressive disorders. Acculturation stress refers to the psychological impact of navigating between two cultures, balancing the preservation of one’s cultural identity with the requirements of adapting to a new cultural environment. This process can lead to feelings of alienation, cultural conflict, and identity confusion, which can subsequently contribute to mental health issues.

The findings are consistent with broader research on acculturation stress, which has shown that migrants facing significant barriers to integration - such as language difficulties, unemployment, and social exclusion - are at a higher risk of developing mental health problems (11, 50–52). Moreover, the study emphasizes the importance of addressing both external stressors (e.g., discrimination, unemployment) and internal conflicts (e.g., identity struggles, unmet expectations) that migrants face during acculturation.

At the same time, some caution is warranted in interpreting generational aspects. The vast majority of participants in our study (over 93%) belonged to the first generation of Turkish migrants, which limits the ability to draw conclusions about the experiences of the second generation. First-generation migrants generally exhibit a higher tendency toward somatization, often linked to cultural, linguistic, and systemic barriers in the host country. On the contrary, second-generation migrants typically benefit from better integration, greater proficiency in the host country language, and a more open attitude towards mental health issues; however, cultural influences and intergenerational conflicts may still affect psychological well-being (15). Due to the small number of second-generation participants in our sample, no subgroup analyses were conducted. Future research should specifically address this issue in more balanced samples.

The study findings have significant implications for the treatment and support of Turkish-speaking migrants in Germany. Given the strong correlation between migration-related stressors and mental health symptoms, it is essential to develop targeted interventions that address both the psychological and practical challenges faced by this population. Healthcare providers should be trained to recognize the cultural factors influencing somatization and to develop culturally sensitive approaches to mental health care. This may include integrating traditional beliefs and practices into therapeutic interventions. Language support is also a key intervention area. The study emphasizes the need for accessible healthcare services in the native language of migrants to ensure better communication between patients and providers. This could involve increasing the availability of Turkish-speaking healthcare professionals or providing interpreter services in healthcare settings. Addressing language barriers will improve mental health outcomes and increase trust and engagement with the healthcare system.

In addition, the emotional and social challenges of migration, such as family separation, loneliness, and discrimination, can have significant impacts on mental health. These effects can be mitigated through social support networks and community integration programs that foster connections among migrants with shared cultural backgrounds and promote social inclusion and a sense of belonging. Consistent with this, Kim et al. (53) found that social support significantly moderated the impact of acculturative stress on depression, highlighting the protective role of supportive networks in alleviating migration-related psychological stress.

In addition to migration-related stressors, it is equally important to consider resilience factors in the context of migration. The current body of research on this topic remains limited. A qualitative data analysis by Walther et al. (54) showed that participants described resilience either as a personal trait or as a lasting inner attitude. Furthermore, voluntary engagement, employment, and activism were experienced as promoting resilience. Five additional themes emerged as resilience-enhancing factors: social support; the perception of migration as an opportunity, both in general and specifically for personal development, the experience of parenthood, and being young. In addition, future orientation, hope, religiosity or spirituality, caring for others, and the creation of new opportunities were also found to support the development of resilience among migrants (27). Our findings suggest that migration-related stressors play a central role in the manifestation of both depressive symptoms and somatization among Turkish patients in psychiatric care. If these stressors are not systematically considered during diagnostic and therapeutic processes, there is a risk that treatment remains insufficient by focusing only on symptom relief without addressing underlying contributing factors. This may partly explain why standard treatment approaches often show limited effectiveness in migrant populations, as they fail to integrate the social, cultural, and migration-specific dimensions that shape patients’ symptomatology. Consequently, a more comprehensive clinical perspective that acknowledges migration-related stress is crucial for improving both diagnostic accuracy and treatment outcomes.

## Limitations

Several limitations must be acknowledged. First, the relatively small sample size (60 participants) restricts the generalizability of the results. While the study offers a snapshot of the mental health challenges faced by Turkish-speaking migrants, a larger and more diverse sample would be necessary to validate these findings across different migrant groups. Additionally, the study’s cross-sectional design does not facilitate causal inferences. Longitudinal studies would be more effective in determining whether migration stressors directly lead to increased rates of depression and somatization over time. Finally, while the study mainly focuses on first-generation migrants, the experiences of second-generation migrants may vary, and future research should investigate intergenerational differences in mental health outcomes.

## Conclusion

Taken together, our findings demonstrate the high prevalence of migration-related stressors, particularly language barriers, social isolation, and disrupted family structures, among migrants treated in a psychiatric hospital. Participants showed elevated levels of both depressive symptoms and somatization, with a strong dose–response relationship between the number of stressors and depression, as well as a significant association between migration-related stressors and somatization. These results underscore the clinical importance of considering migration-related stress in psychiatric assessment and treatment, in order to avoid insufficient care that overlooks key factors in the development of depression and somatization among Turkish patients.

## Data Availability

The raw data supporting the conclusions of this article will be made available by the authors, without undue reservation.
